# Dissemination of Antimicrobial Resistance in Microbial Ecosystems through Horizontal Gene Transfer

**DOI:** 10.3389/fmicb.2016.00173

**Published:** 2016-02-19

**Authors:** Christian J. H. von Wintersdorff, John Penders, Julius M. van Niekerk, Nathan D. Mills, Snehali Majumder, Lieke B. van Alphen, Paul H. M. Savelkoul, Petra F. G. Wolffs

**Affiliations:** ^1^Department of Medical Microbiology, NUTRIM School for Nutrition and Translational Research in Metabolism, Maastricht University Medical Center+Maastricht, Netherlands; ^2^Department of Medical Microbiology, Caphri School for Public Health and Primary Care, Maastricht University Medical Center+Maastricht, Netherlands; ^3^Department of Medical Microbiology and Infection Control, VU University Medical CenterAmsterdam, Netherlands

**Keywords:** antibiotic resistance, resistome, transformation, conjugation, transduction, gene transfer agents, GTA, lateral gene transfer

## Abstract

The emergence and spread of antibiotic resistance among pathogenic bacteria has been a rising problem for public health in recent decades. It is becoming increasingly recognized that not only antibiotic resistance genes (ARGs) encountered in clinical pathogens are of relevance, but rather, all pathogenic, commensal as well as environmental bacteria—and also mobile genetic elements and bacteriophages—form a reservoir of ARGs (the resistome) from which pathogenic bacteria can acquire resistance via horizontal gene transfer (HGT). HGT has caused antibiotic resistance to spread from commensal and environmental species to pathogenic ones, as has been shown for some clinically important ARGs. Of the three canonical mechanisms of HGT, conjugation is thought to have the greatest influence on the dissemination of ARGs. While transformation and transduction are deemed less important, recent discoveries suggest their role may be larger than previously thought. Understanding the extent of the resistome and how its mobilization to pathogenic bacteria takes place is essential for efforts to control the dissemination of these genes. Here, we will discuss the concept of the resistome, provide examples of HGT of clinically relevant ARGs and present an overview of the current knowledge of the contributions the various HGT mechanisms make to the spread of antibiotic resistance.

## Introduction

The ever-increasing magnitude of antimicrobial resistance (AMR) encountered in human pathogens is a huge concern for public health worldwide, limiting treatment options for bacterial infections and thereby reducing clinical efficacy while increasing treatment costs and mortality. With a lack of development of new antibiotics, and increasing resistance even to last-resort antibiotics (Nordmann et al., 2012), there is a need to conserve the ones available.

Natural antibiotics have existed for billions of years (Barlow and Hall, 2002; Hall and Barlow, 2004; Bhullar et al., 2012; Wright and Poinar, 2012), providing a selective benefit for the producing strains by inhibiting or eliminating other bacteria competing for resources (Martinez, 2008; Aminov, 2009). Additionally, their function as cell-cell signaling molecules has been described (Davies, 2006; Linares et al., 2006). Just as antibiotics are ancient, so are antibiotic resistance genes (ARGs), as evidenced by studies identifying various ARGs in ancient permafrost samples (D'Costa et al., 2011; Perron et al., 2015) and isolated cave microbiomes (Bhullar et al., 2012). Resistance to antibiotics can occur either by mutations or by acquisition of resistance conferring genes via horizontal gene transfer (HGT), of which the latter is considered to be the most important factor in the current pandemic of AMR.

The HGT of ARGs also far predates the production and use of antibiotics by humans. For example, OXA-type β-lactamases were found to be plasmid-borne and able to transfer between bacterial species millions of years ago (Barlow and Hall, 2002). However, while antibiotic resistance and its spread by HGT are ancient mechanisms, the rate at which these processes occur and the number of resistant strains has increased tremendously over the past few decades because of selective pressure through human antibiotic use.

## The global antibiotic selection pressure

While the discovery of antibiotics revolutionized the field of medicine, their increasingly large-scale production and consumption has had widespread effects on the microbial biosphere. In their analysis, Van Boeckel et al. showed that global human antibiotic consumption amounted to 54,083,964,813 standard units (pills, capsules, or ampoules) in 2000 and had increased by 36% to 73,620,748,816 standard units by 2010 (Van Boeckel et al., 2014). The same authors estimated that antibiotic consumption in food animals, which is assumed to be larger than that of humans, was over 63 million kg in 2010 and will also drastically increase in the coming years (Van Boeckel et al., 2015) despite recent initiatives to reduce antibiotic use in animals. Such high and continuously increasing amounts of antibiotics overwhelm the natural production, causing a constantly increasing selection pressure on bacterial populations in all exposed environments.

The use and misuse of antibiotics in medicine, agriculture, and aquaculture has been linked to the emergence of resistant bacteria in these settings (Cabello, 2006; Penders and Stobberingh, 2008; Economou and Gousia, 2015). However, the impact of antibiotic usage extends further, as antibiotic residues, resistant bacteria, and genetic resistance elements subsequently spread to adjacent environments. The majority of consumed antibiotics are excreted unchanged (Sarmah et al., 2006) and are then introduced into the environment directly or through waste streams. Such waste streams, as well as wastewater treatment plants, are considered to be hotspots for the dissemination of AMR, since resistance genes, mobile genetic elements (MGEs), and (sub-inhibitory) antibiotic selection pressure from various sources are introduced to commensals and pathogens (Tennstedt et al., 2003; Martinez, 2009; Zhang et al., 2009; Graham et al., 2011). Moreover, the antibiotic compounds are often not completely removed in treatment plants (Watkinson et al., 2007; Le-Minh et al., 2010), from where they then disseminate further. A study by Larsson et al. showed that a treatment plant in India, receiving water from drug manufacturing sites, exposes its direct environment to very high levels of antibiotics, discharging ~45 kg of ciprofloxacin per day (Larsson et al., 2007), contaminating surface, ground, and drinking water in the area (Fick et al., 2009). As a result, not only were highly multiresistant bacteria found to be common within the treatment plant (Marathe et al., 2013), but high levels of ARGs and MGEs were also detected in nearby river sediments (Kristiansson et al., 2011).

How environmental exposure to antibiotics contributes to the selection of resistant strains and the increase of resistome elements is illustrated by a study comparing soil samples taken between 1940 and 2008, which shows that ARGs from all classes of antibiotics tested (β-lactams, tetracyclines, erythromycins, and glycopeptides) significantly increased since 1940, with a tetracycline ARG being over 15 times more abundant than in the 1970s (Knapp et al., 2010). Moreover, the increasing selection pressure has altered bacterial HGT processes, increasing the number of resistome elements which reside on mobile DNA compared to the pre-antibiotic era (Datta and Hughes, 1983).

## Reservoirs of resistance

In order to understand the dissemination of antibiotic resistance, it is necessary to map the resistome of various environments, and to unravel to what extend these environments can act as a reservoir for the dissemination of ARGs to bacterial pathogens. In recent years there has been increasing interest in this matter, as many studies have used various techniques to sample the resistome of environments such as, but not limited to, soil, wastewater, and human and animal gut microbiota (Pehrsson et al., 2013; Penders et al., 2013; Rizzo et al., 2013; von Wintersdorff et al., 2014). It has since become clear that ARGs, including clinically relevant ones, are widespread in such environments (Wright, 2010). Studies applying a metagenomic approach directly recover DNA from all micro-organisms in a biological sample, thereby avoiding the bias that is introduced when selecting certain organisms, and allowing for the investigation of the resistome of microbial ecosystems. The sequencing of metagenomes from various environments has led to a wealth of data which is often publicly available in databases. Such databases can be mined for the presence of resistance genes, even when the initial studies did not focus specifically on ARG content in these metagenomes. For example, mining such metagenomic databases for the plasmid-mediated colistin resistance gene *mcr-1*, which has recently been discovered in clinical and commensal isolates (Arcilla et al., 2015; Liu et al., 2015), has revealed that this gene had already spread to the human gut microbiome of Chinese subjects several years ago (Hu et al., 2015). While sequence based studies provide huge amounts of data, they are limited to either identifying genes that are already known, or to predicting novel sequence functions based on high homology to known sequences. Annotation by sequence-based studies will keep increasing however, as studies using functional metagenomics keep identifying novel ARGs. An increasing number of such studies have revealed a huge number of previously unknown ARGs present in environments such as soil (Riesenfeld et al., 2004; D'Costa et al., 2006; Allen et al., 2009; Donato et al., 2010; Torres-Cortes et al., 2011; Perron et al., 2015) or activated sludge (Mori et al., 2008; Parsley et al., 2010), as well as in the microbiota of animals (Kazimierczak et al., 2009; Wichmann et al., 2014) and humans (Sommer et al., 2009; Cheng et al., 2012; Moore et al., 2013, 2015; Card et al., 2014; Fouhy et al., 2014; Clemente et al., 2015).

Recent metagenomic studies have also uncovered that ARGs predominantly cluster by ecology, implying that the resistome in soils, and wastewater treatment plants differ significantly from that of human pathogens (Gibson et al., 2015; Munck et al., 2015). Nonetheless, the authors of these works note that parts of these resistomes are shared (Forsberg et al., 2012) and stress the importance of continuing the exploration of the resistome of such environments.

That commensals and the environment are important reservoirs for resistance is supported by several examples of ARGs on MGEs in human pathogens that appear to have originated from those reservoirs. A well-known example is that of the *bla*_CTX−M_ genes, which have become the most prevalent cause of extended-spectrum β-lactamases (ESBLs) in Enterobacteriaceae worldwide and a major cause of clinical treatment problems (Hawkey and Jones, 2009). The potential origin of these genes was identified as the chromosomal DNA of various environmental *Kluyvera* species, from where they spread very successfully to different bacterial species (Canton and Coque, 2006). *Shewanella algae*, a marine and freshwater species, was found to be the origin of plasmid-encoded *qnrA* genes, conferring quinolone resistance (Poirel et al., 2005a), and different *Vibrionaceae* species might be the reservoir for other plasmid-encoded *qnr* genes (Poirel et al., 2005b), which have disseminated globally in various Enterobacteriaceae species, with exceptionally high prevalence rates in some areas (Vien le et al., 2012). The OXA-48-type carbapenem-hydrolyzing β-lactamase genes, which are increasingly reported in enterobacterial species worldwide, were also found to originate from the chromosomes of waterborne, environmental *Shewanella* species (Poirel et al., 2012). As with these few examples, many clinically relevant resistance genes are believed to have originated from non-pathogenic bacteria, highlighting the immense potential of HGT for these pathogens in overcoming human use of antibiotics.

## Contribution of the various HGT mechanisms to the spread of ARGs

### Conjugation

**Figure 1:**
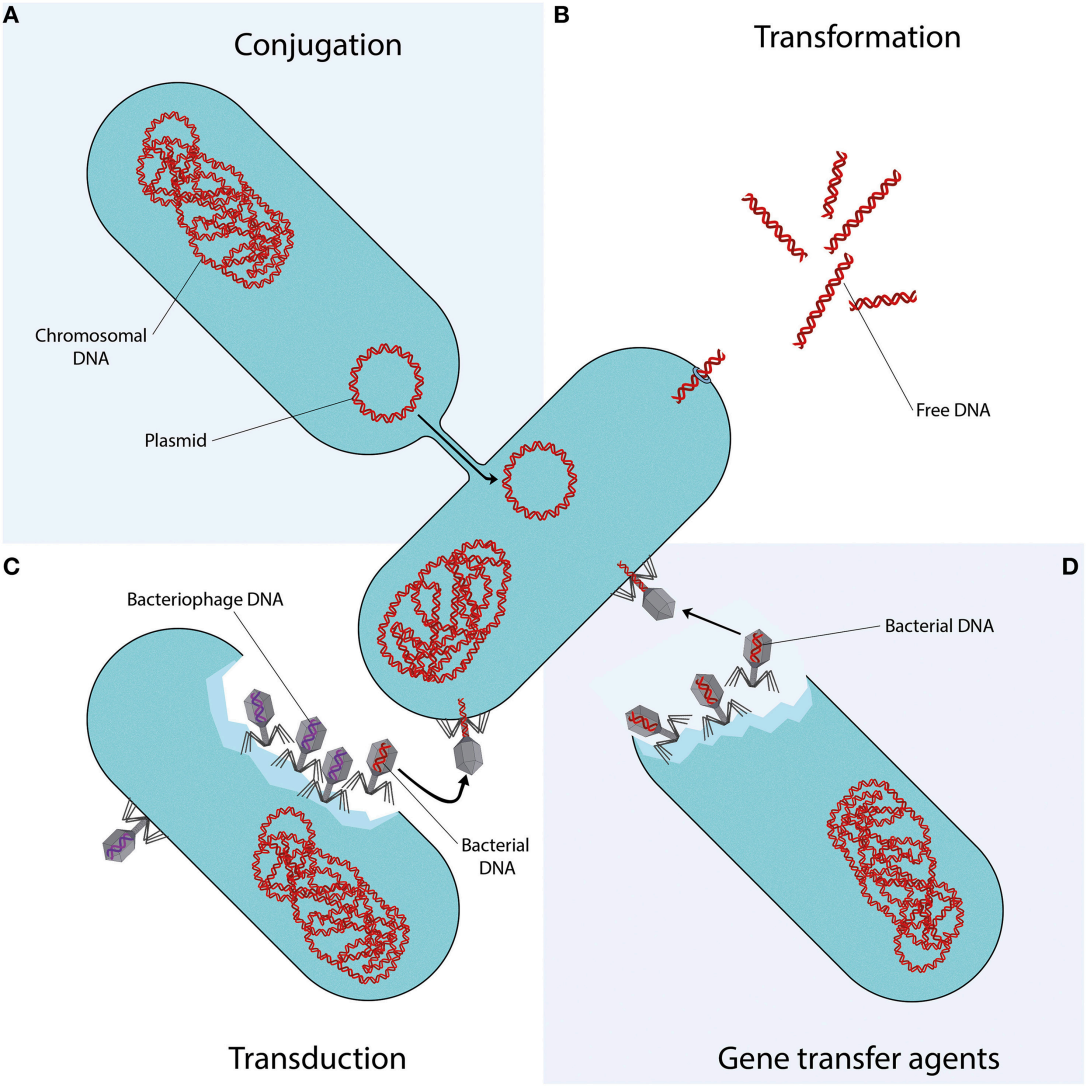
**Mechanisms of horizontal gene transfer**. Each quadrant represents one different method of gene transfer. **(A)** Conjugation is a process requiring cell to cell contact via cell surface pili or adhesins, through which DNA is transferred from the donor cell to the recipient cell. **(B)** Transformation is the uptake, integration, and functional expression of naked fragments of extracellular DNA. **(C)** Through specialized or generalized transduction, bacteriophages may transfer bacterial DNA from a previously infected donor cell to the recipient cell. During generalized transduction, bacterial DNA may be accidentally loaded into the phage head (shown as a phage with a red DNA strand). During specialized transduction, genomic DNA neighboring the prophage DNA is co-excised and loaded into a new phage (not shown). **(D)** Gene transfer agents (GTAs) are bacteriophage-like particles that carry random pieces of the producing cell's genome. GTA particles may be released through cell lysis and spread to a recipient cell.

Conjugation is the transfer of DNA through a multi-step process requiring cell to cell contact via cell surface pili or adhesins. It is facilitated by the conjugative machinery which is encoded either by genes on autonomously replicating plasmids or by integrative conjugative elements in the chromosome (Smillie et al., 2010; Wozniak and Waldor, 2010). Additionally, this conjugative machinery may enable the mobilization of plasmids that are non-conjugative, as observed for e.g., the exceptionally broad host range IncQ plasmids (Meyer, 2009). Of the various mechanisms that may facilitate HGT (Figure 1), conjugation is certainly the most commonly studied (Norman et al., 2009; Guglielmini et al., 2013).

ARGs are in many cases associated with conjugative elements such as plasmids or transposons. While the transfer of these elements may also occur through transformation or transduction, conjugation is often considered as the most likely responsible mechanism. This is due to the fact that it provides better protection from the surrounding environment and a more efficient means of entering the host cell than transformation, while often having a broader host range than bacteriophage transduction. Moreover, while conjugation is a process directed toward the transfer of bacterial genes, transfer of bacterial DNA by transduction is a side-effect of erroneous bacteriophage replication (Norman et al., 2009).

The conjugation of MGEs conferring AMR has been observed in many types of ecosystems, ranging from transfer between bacteria in insects, soil, and water environments to various food and healthcare associated pathogens (Davison, 1999). Importantly, transfer of plasmids and conjugative transposons, such as those of the Tn916 family, between unrelated bacteria over large taxonomic distances has been described (Shoemaker et al., 2001; Musovic et al., 2006; Roberts and Mullany, 2009; Tamminen et al., 2012), indicating that this mechanism contributes significantly to the dissemination of ARGs between different reservoirs via such broad host range MGEs.

The spread of antibiotic resistance plasmids in human pathogens is especially well studied, and shows that once resistance genes have become established on successful plasmids, they may rapidly spread across different strains, species, or even genera. This is well demonstrated by the *bla*_CTX−M_ ESBL genes, which have disseminated to various narrow and broad host range plasmids within Enterobacteriaceae, as well as to other opportunistic human pathogens (Canton et al., 2012). These genes are now ubiquitous in humans (Woerther et al., 2013), animals, and the environment (Hartmann et al., 2012). Furthermore, the transfer of plasmids in pathogens has led to the worldwide spread of numerous ARGs encoding resistance to β-lactams, quinolones, aminoglycosides, tetracyclines, sulfonamides, and many other drug classes (Huddleston, 2014). Of particular concern is the increasingly reported spread of plasmids harboring carbapenem resistance (Carattoli, 2013) and the recent discovery of plasmid-encoded colistin resistance in China (Liu et al., 2015), which has now already been identified at multiple continents (Arcilla et al., 2015) and may cause Enterobacteriaceae to truly become pan-drug resistant. Moreover, multiple ARGs are often co-localized on the same plasmid, which allows for the relatively easy spread of multidrug resistance.

### Transformation

In 1928, Griffith became the first to demonstrate direct genetic exchange between different strains of *Streptococcus pneumoniae* (Griffith, 1928). Certain bacteria appeared to be capable of uptake, integration, and functional expression of naked fragments of extracellular DNA, a process called (natural) transformation. It soon became clear that bacteria could use transformation to evade antibiotics, by exchanging ARGs. In 1951, Hotchkiss induced penicillin and streptomycin resistance in previously sensitive strains of *S. pneumoniae* by exposing them to DNA from resistant strains (Hotchkiss, 1951). Alexander et al. furthered this work by demonstrating intra- and inter-species transfer of streptomycin resistance between *H. influenzae, H. parainfluenzae*, and *H. suis* (Alexander and Leidy, 1953; Alexander et al., 1956).

In order for transformation to take place, several conditions have to be met. There must be DNA present in the extracellular environment; the recipient bacteria must be in a state of competence; and the translocated DNA must be stabilized, either by integration into the recipient genome, or by recircularisation (in the case of plasmid DNA) (Thomas and Nielsen, 2005). Whereas Neisseria spp. are considered to be constitutively competent (Sparling, 1966; Johnston et al., 2014), other bacterial species capable of natural transformation may develop competence only under certain conditions, such as the presence of peptides or autoinducers, nutritional status, or other stressful conditions, as reviewed in more detail by Johnston et al. (2014). Importantly, studies have shown that exposure to antibiotics can induce competence in many species of bacteria, meaning that antibiotics would not only select for resistant strains, but also stimulate transformation of their ARGs (Prudhomme et al., 2006; Charpentier et al., 2011, 2012).

*In vitro* experiments have done much to elucidate transformation of ARGs. Early work proved that ARGs could be transformed; to this end, streptomycin, rifampicin, erythromycin, nalidixic acid, and kanamycin resistance have variously been transformed into *Neisseria gonorrhoeae* (Sparling, 1966), *Bacillus* spp. (Harford and Mergeay, 1973), *Gallibacterium anatis* (Kristensen et al., 2012), and *S. pneumoniae* (Prudhomme et al., 2006). The introduction of molecular techniques allowed for the identification of the ARGs being transformed. *In vitro* studies have shown that the genes *parC* and *gyrA* are involved in the transformation of fluoroquinolone resistance between *S. pneumoniae* (Ferrandiz et al., 2000) and several viridans streptococci (Gonzalez et al., 1998; Janoir et al., 1999), and that transformation of *penA* confers penicillin resistance in commensal *Neisseria* species (*N. flavescens* and *N. cinerea*) and *N. meningitidis* (Bowler et al., 1994).

Molecular techniques have also made it possible to look for evidence of transformation outside of the laboratory. Spratt et al. identified the *penA* variant responsible for penicillin resistance in clinical isolates of *N. gonorrhoeae* (Spratt, 1988); sequence analysis revealed a mosaic structure, with blocks homologous to susceptible-type *penA* and blocks that diverge significantly (Spratt et al., 1992). These “resistant blocks” could be traced back to a strain of *N. flavescens* that had been isolated in the pre-antibiotic era, suggesting that such commensal species could have been the original source for the now ubiquitous resistance to penicillin (Spratt et al., 1989; Lujan et al., 1991). Mosaic genes are formed when sections of foreign DNA are incorporated into a recipient genome, as is the case in transformation. Their presence implies that HGT has taken place (Hakenbeck, 1998). In streptococci, the mosaic penicillin-binding protein (PBP) genes that encode PBPs with decreased affinity for β-lactam antibiotics are believed to be the result of gene transfer from related penicillin-resistant species (Sibold et al., 1994) and have disseminated penicillin resistance between various streptococci species (Dowson et al., 1990). Studies of fluoroquinolone resistance have demonstrated that mosaic variants of the genes *parC, parE*, and *gyrA* are readily transformed between *S. pneumoniae, Streptococcus mitis* and *Streptococcus oralis* (Balsalobre et al., 2003), and between *Streptococcus pyogenes* and *Streptococcus dysgalactiae* (Pletz et al., 2006).

Mao et al. developed a technique to extract intra- and extracellular DNA separately from environmental samples, and applied it to samples from a river basin in China. The result—a greater abundance of DNA outside of cells than inside—implies that in certain environments, extracellular DNA is a large reservoir for genes which may be accessible via transformation (Mao et al., 2014). Furthermore, Domingues et al. demonstrated that MGEs such as transposons, integrons and gene cassettes can be disseminated efficiently between species, regardless of their level of genetic relatedness (Domingues et al., 2012). Similarly, streptococcal species have been shown to exchange conjugative transposons via transformation in addition to conjugation (Chancey et al., 2015). All of this indicates that transformation provides a broad capacity for the horizontal spread of resistance elements between divergent species.

### Transduction

Bacteriophages play an important role in shaping the bacterial microbiome in any environment. Through specialized or generalized transduction, bacteriophages can transfer genes that are advantageous to their microbial hosts, in turn promoting their own survival and dissemination (Modi et al., 2013). The transferable DNA sequences range from chromosomal DNA to MGEs such as plasmids, transposons and genomic islands (Brown-Jaque et al., 2015).

The mobilization or transfer of ARGs by bacteriophages has been documented for various bacterial species: the transduction of erythromycin (Hyder and Streitfeld, 1978), tetracycline or multiple resistances between strains of *S. pyogenes* (Ubukata et al., 1975); the transfer of tetracycline and gentamicin resistance between enterococci (Mazaheri Nezhad Fard et al., 2011); the carriage of β-lactamase genes by bacteriophages in *Escherichia coli* (Billard-Pomares et al., 2014) and *Salmonella* (Schmieger and Schicklmaier, 1999); or the transfer of antibiotic resistance plasmids in MRSA (Varga et al., 2012).

Recent studies applying metagenomic approaches to samples from various environments have suggested that bacteriophages may play a bigger part in the spread of ARGs than previously recognized. Colomer-Lluch et al. used qPCR to show that the β-lactam ARGs *bla*_TEM_, *bla*_CTX−M_ and *mecA* were present in bacteriophages from river and urban sewage water samples. Additionally, cloning of the phage DNA into ampicillin susceptible *E. coli* hosts resulted in resistant transformants, harboring either the *bla*_TEM_, *bla*_CTX−M_, or undetermined ARGs (Colomer-Lluch et al., 2011a). In another study, the presence of ARGs in bacteriophages was detected in respiratory tract DNA of cystic fibrosis patients (Rolain et al., 2011). Modi et al. demonstrated that treatment with antibiotics increased the number of ARGs in the intestinal phageome of mice and expanded the interactions between phage and bacterial species (Modi et al., 2013), which is an important observation considering the increased environmental exposure to antibiotics discussed earlier. Furthermore, several studies have used qPCR to detect ARGs in bacteriophages from wastewater samples (Colomer-Lluch et al., 2014a,b), animal and human fecal samples (Colomer-Lluch et al., 2011b; Quiros et al., 2014), wastewater and sludge derived from wastewater treatment plants (Calero-Caceres et al., 2014), and hospital and wastewater treatment plant effluents (Marti et al., 2014), indicating that bacteriophages are significant reservoirs of ARGs. Shousha et al. investigated bacteriophages isolated from chicken meat and found that about a quarter of the randomly isolated bacteriophages were able to transduce resistance to one or more antibiotics into an *E. coli* host. Moreover, they found a significant relationship between the presence of bacteriophages transducing kanamycin ARGs, and *E. coli* isolates resistant to kanamycin, implying a possible role of this mechanism in the spread of AMR (Shousha et al., 2015).

Considering certain bacteriophages have been reported to have a wide host range that crosses between different species (Mazaheri Nezhad Fard et al., 2011) or even different taxonomic classes (Jensen et al., 1998), the observation of the plethora of ARGs carried by bacteriophages in various bacterial communities and environments provides renewed insights into the role of transduction in the dissemination of ARGs in microbial ecosystems.

### Gene transfer agents

Gene transfer agents (GTAs), first identified in *Rhodobacter capsulatus* (RcGTA) in 1974 (Marrs, 1974), are host-cell produced particles that resemble bacteriophage structures, capable of transferring genetic content. GTAs have several characteristic features: (i) rather than carrying DNA encoding their own machinery (as with self-propagating bacteriophages), GTAs carry random pieces of the producing cell's genome (Marrs, 1974; Humphrey et al., 1997; Stanton, 2007; Hynes et al., 2012); (ii) the amount of DNA packaged by the GTAs is insufficient to encode all of their protein components, making them unable to self-propagate (Lang and Beatty, 2000, 2001, 2007); (iii) GTA production is controlled by cell regulatory mechanisms (Lang and Beatty, 2000; Leung et al., 2012; Mercer et al., 2012; Brimacombe et al., 2014); (iv) GTA particles are released through cell lysis (Hynes et al., 2012; Westbye et al., 2013) although cultures do not display observable lysis (Marrs, 1974) as only a small subpopulation of GTA-producing cultures (~3%) is responsible for ~95% of GTA release (Fogg et al., 2012; Hynes et al., 2012); (v) recently, it has been proposed that GTAs combine key aspects of transduction and transformation for cell entry, requiring proteins involved in natural transformation (Brimacombe et al., 2015).

Although GTA particles do not necessarily carry any GTA-encoding genes (Lang et al., 2012), RcGTA-like gene clusters are widespread in alphaproteobacteria, especially in the *Rhodobacterales*: a complete set of RcGTA-like structural genes has been demonstrated in nearly every sequenced member of the *Rhodobacterales* (Lang and Beatty, 2007; Lang et al., 2012). Moreover, two species in the order of *Rhodobacterales, Roseovarius nubinhibens* and *Ruegeria mobilis*, are known to produce GTAs, and there is evidence of GTA production in *Ruegeria pomeroyi* (Biers et al., 2008; McDaniel et al., 2010; Lang et al., 2012). Other known GTAs are VSH-1 in the spirochaete *Brachyspira hyodysenteriae*, Dd1 in the deltaproteobacterium *Desulfovibrio desulfuricans*, and VTA in the archaeon *Methanococcus voltae* (Lang and Beatty, 2007; Lang et al., 2012). The genes required for GTA-production are contained within the host genome and appear to have been propagated through vertical transmission (Lang and Beatty, 2007).

It has been suggested that GTAs have several advantages over the previously described mechanisms of HGT (Stanton, 2007): GTA particles afford DNA protection from damaging environmental factors, as opposed to the naked DNA involved in natural transformation; compared to conjugation, the transfer ability of GTAs is likely maintained after conditions killing the host cell, and is moreover not constrained by cell-to-cell contact; lastly, compared to transduction, GTA particles predominantly carry random pieces of host genome, rather than mostly bacteriophage DNA. In the marine environment, GTA-mediated transfer events have been reported to be remarkably high; up to several million times higher than previous estimates of HGT in marine environments, exceeding previously described transformation and transduction frequencies (McDaniel et al., 2010). Moreover, genes can be exchanged between bacterial phyla (McDaniel et al., 2010; Lang et al., 2012), suggesting the possible widespread contribution of GTAs in shaping and driving adaptation of the natural environment.

In culture, GTA mediated transfer of antibiotic resistance markers has been readily demonstrated in *R. capsulatus* (Marrs, 1974; Solioz et al., 1975; Wall et al., 1975) and the spirochaete *Brachyspira hyodesenteriae* (Stanton et al., 2001, 2008). Moreover, GTAs have been used to transfer traits from plasmids (Scolnik and Haselkorn, 1984). In addition, the *B. hyodesenteriae* GTA VSH-1 can be induced by certain antibiotics (Stanton et al., 2008), which points out its possible impact in its natural environment, the swine intestinal tract. Other *Brachyspira* spp. occur in the intestinal tract of other species, including humans and chickens (Hampson and Ahmed, 2009), in which VSH-1 genes have been described (Motro et al., 2009). However, interspecies GTA-mediated transfer remains to be demonstrated (Motro et al., 2009).

The impact of GTAs on human health has yet to be established, but given the high frequency of transfer events in certain environments, and their ability to exchange genes between phyla, their potential to act as vehicles of resistance traits in the environment, and within the microbiota of humans and farmed animals is an area worthy of further study.

## Conclusion

The increase in environmental levels of antibiotics, driven by medical and agricultural demand, is unprecedented and has disrupted the natural balance between microbes and antimicrobials. The effects this has on microbial communities are wide-ranging, and the result is an increasingly tangible threat to healthcare, as resistance to all known antibiotics disseminates rapidly around the globe. Our knowledge of the interactions between antimicrobials and resistance against it, observed not only in the clinic but across different ecosystems around the world, is rapidly increasing and has provided valuable insights. However, it is vital that we continue to unravel the extent of, and dissemination between resistomes of these microbial ecosystems, as any attempt at coming to terms with the AMR problem will have to account for these vast reservoirs of ARGs.

## Author contributions

CW, JN, SM wrote the article. CW, JN made the Figure. JP, LA, PS, PW provided feedback and discussion on the article.

### Conflict of interest statement

The authors declare that the research was conducted in the absence of any commercial or financial relationships that could be construed as a potential conflict of interest.
